# Thionation of Some α,β-Unsaturated Steroidal Ketones

**DOI:** 10.3390/molecules15053462

**Published:** 2010-05-12

**Authors:** Natalija M. Krstić, Mira S. Bjelaković, Milan M. Dabović, Vladimir D. Pavlović

**Affiliations:** 1 Center for Chemistry, Institute of Chemistry, Technology and Metallurgy, University of Belgrade, Studentski trg 12-16, P. O. Box. 473, 11001 Belgrade, Serbia; E-Mails: mbjelak@chem.bg.ac.rs (M.S.B.); mdabovic@chem.bg.ac.rs (M.M.D.); 2 Faculty of Chemistry, University of Belgrade, Student ski trg 12-16, P. O. Box 158, 11001 Belgrade, Serbia; E-Mail: pavlovic@chem.bg.ac.rs (V.D.P.)

**Keywords:** α,β-unsaturated steroidal ketones, α,β-unsaturated 3-thiones, Lawesson’s reagent, P_4_S_10_/HMDO, dimerization, microwave irradiation

## Abstract

The reactions of selected α,β-unsaturated steroidal ketones with Lawesson’s reagent (LR) in CH_2_Cl_2_ and toluene under the standard reaction conditions and with a combination of phosphorus pentasulfide with hexamethyldisiloxane (P_4_S_10_/HMDO) in 1,2-dichlorobenzene (ODCB) under microwave irradiation were investigated and for this purpose several cholestane, androstane and pregnane carbonyl derivatives were chosen. Depending on the reagent and the solvent, 19 new sulfur containing compounds, including dithiones **4c** and **4d**, α,β-unsaturated 3-thiones **3a**-**e**, dimer-sulfides **2a**-**e**, 1,2,4-trithiolanes **5a-e** and phosphonotrithioates **6b-e** were synthesized. All newly prepared compounds were characterized by IR, ^1^H- and ^13^C-NMR spectroscopy and elemental analysis.

## 1. Introduction 

Steroids are an important group of natural compounds possessing a variety of biological activities. The replacement of one or more carbon atoms in a steroid molecule by a heteroatom affects the chemical properties of that particular steroid and often results in alterations of its biological activity, which sometimes may be useful. Also, the addition of heterocyclic rings or new functional groups to steroid molecules often leads to changes in their physiological activity. Thionation is one possible modification that could have some influence on such activity. The larger and less electronegative sulfur atom, relative to oxygen, might alter the hydrogen-bonding ability and/or induce conformational changes in the modified molecule. In addition the increased reactivity of the thione function should make these derivatives useful intermediates for further transformations. Recently we reported synthesis of 6-thioxo-7-aza-B-homocholest-4-ene and 6-aza-7-thioxo-B-homocholest-4-ene using Lawesson’s reagent [1]. Continuing this investigation and our previous work on modified steroid compounds as biologically active molecules [1,2,3,4], the goal of this study was to synthesize some new thioxosteroid derivatives.

**Figure 1:**
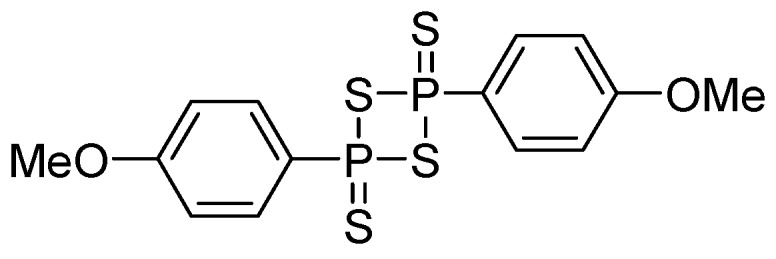
Lawesson’s reagent.

Several methods are reported in the literature for the thionation of organic compounds. Phosphorus pentasulfide (P_4_S_10_) was first reported in 1869 by Henry [5] and Wislicenus [6]. The usual procedure, which involves boiling toluene, xylene or pyridine as solvent, requires a large excess of reagent, long reaction timed, and results in low and variable yields [7,8,9]. Recently Curphey [10,11,12] has shown that a combination of P_4_S_10_ with hexamethyldisiloxane (HMDO) efficiently converts esters, lactones, amides and ketones into their corresponding thio derivatives. Under the standard reaction conditions (dry toluene/xylene, thermal heating) this method has provided an increase in the selectivity and yield. Kaushik *et al*. developed a new thionating reagent by encapsulating the P_4_S_10_ in basic alumina [13]. The reactions were carried out in good to excellent yields by refluxing mixture of ketone and P_4_S_10_/Al_2_O_3_ in acetonitrile, but this method needs some more investigation. In 1978, Lawesson and coworkers developed a new reagent, 2,4-bis(*p*-methoxyphenyl)-1,3-dithiadiphosphetane-2,4-disulfide, commonly named Lawesson’s reagent (LR, Figure 1) [14,15,16]. 

The attractiveness of LR is associated with its commercial availability, simplicity and convenience of use, high yields and especially soft thionation reactions. In some cases the main disadvantage of this reagent is the formation of byproducts or stable heterocyclic intermediates. However, some of these sulfur and phosphorus containing products can be very useful as they have showed a promising anti-microbial and anti-inflammatory activity [17,18,19,20]. Since all described procedures require the use of dry aromatic hydrocarbon solvents and lengthy reaction times, microwave assistance has been applied to thionate a wide variety of substrates [21,22,23,24,25].

α,β-Unsaturated thiones are relatively little known, presumably due to their instability as monomeric species. Thus, the conversion of α,β-unsaturated ketones into corresponding thiones remains a challenging problem for organic chemists. Thionation of some steroidal ketones is already described in literature. Barton and coworkers synthesized several Δ^1,4^-diene-3-thioxo corticosteroids using P_4_S_10_ in pyridine as a reagent [26]. Weis *et al*. also described the regioselective thionation of 3-oxo-1,4-diene steroid systems with LR in anhydrous THF [27]. Although several simple alicyclic α,β-unsaturated thiones have been prepared, 3-thioxocholest-4-ene [28], 17-thioxoandrost-4-en-3-one [19] and 3,17-dithioxoandrost-4-ene [19] remain the only characterized steroidal examples. As far as we know there is no report dealing with the synthesis of thiono analogues of steroidal ketones under the microwave irradiation. 

**Figure 2:**
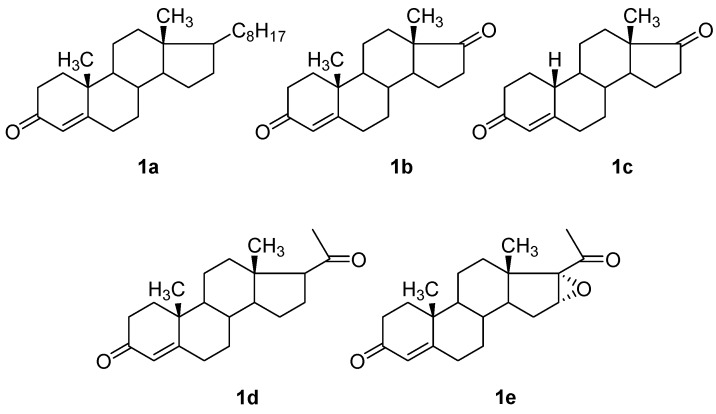
Substrates **1a-e**.

Herein we report on the reaction of several α,β-unsaturated cholestane, androstane and pregnane carbonyl derivatives **1a-e** (Figure 2) with LR (in CH_2_Cl_2_ and toluene) under the standard reaction conditions and with a combination of P_4_S_10_/HMDO in 1,2-dichlorobenzene under microwave irradiation.

## 2. Results and Discussion 

The reactions of α,β-unsaturated ketones **1a-e** with LR gave different products, depending on the solvent and duration of the reaction. Due to the instability of the unsaturated thioketones or dimers formed and their easy reconversion to the starting compounds a certain amount (8–52%) of the parent compounds was isolated in all cases.

**Scheme 1:**
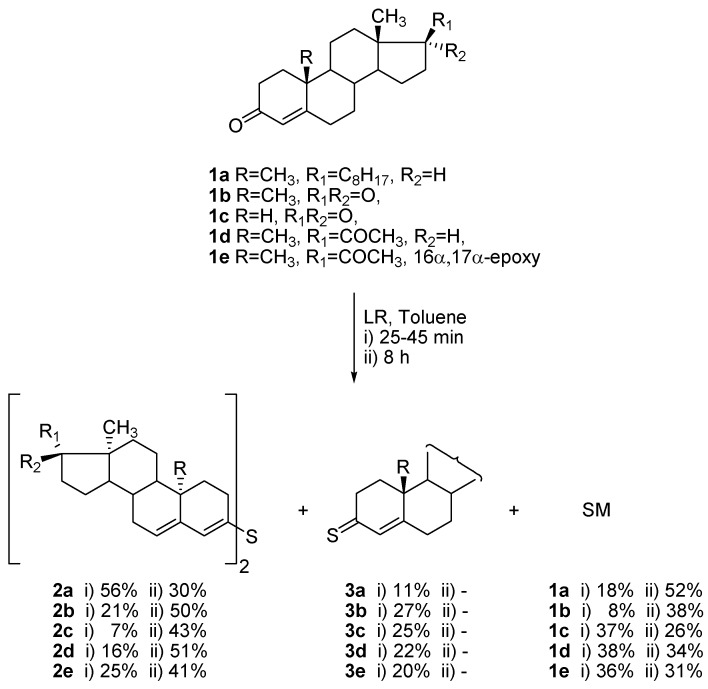
Thionation of **1a**-**e** with LR in toluene.

At the beginning, the reaction procedure for thionation was carried out in refluxing toluene. Instantaneous reaction took place (the purple color of reaction mixture) with simultaneous formation of thionated products. Although there was no complete consumption of **1a**-**e**, the reaction was quenched after 25-45 min (depending on substrate, see Experimental), while the solution was still purple, which was the evidence of the presence of thioketones. After flash column chromatography, besides corresponding dimer-sulfides **2a-e** (7–56%), thioketones **3a**-**e** were isolated in 11–27% yields (Scheme 1). When the reaction procedure was carried out in refluxing toluene for 8 h initially formed unstable α,β-unsaturated thioketones either dimerize to give corresponding dimer-sulfides **2a-e** (30–51%), or decompose to a more stable starting ketones **1a**-**e** (26–52%) (Scheme 1).

All obtained thioketones **3a-e** were pink oils and their structures were determined on the basis of their spectral data (IR, ^1^H-NMR, ^13^C-NMR). In the IR spectra the absorption for the original unsaturated 3-oxo group, was missing, as well as the singlet for C-3 in the ^13^C-NMR spectra at about 199 ppm. Instead, the new singlet at δ 236.7, 237.0, 237.8, 236.9 and 237.4 ppm for C(3)=S, for compounds **3a-e,** respectively, appeared. In the ^1^H-NMR spectra signal for H-4 at δ 6.63, 6.69, 6.74, 6.66 and 6.63 ppm was situated downfield comparing to the resonance for the corresponding proton in starting compounds **1a-e** (at δ 5.72, 5.74, 5.84, 5.80 and 5.70 ppm, respectively). In addition, in all thioketones, both protons at C-2 (α-position to the C=S group) resonate well-separated downfield from the other ring protons due to the strong C=S anisotropy, H-equatorial as a *td* at about δ 3.15 ppm, H-axial as a *dtd*, at about δ 2.50 ppm both with corresponding *J*_HH_ coupling constants.

In the IR spectra of **2a**-**e** the absorptions for the original unsaturated 3-oxo groups at 1667, 1665, 1667, 1665 and 1661 cm^-1^ for **1a-e**, respectively, were missing, as well as the singlet for C-3 in the ^13^C-NMR spectra at about δ 199 ppm. Instead, the new C-3 singlet at δ 129.4, 129.5, 129.5, 129.3, 129.4 ppm and the C-6 doublet at δ 124.3, 123.3, 124.8, 123.8 and 123.6 ppm for compounds **2a-e,** respectively, appeared indicating the formation of a new double bond and Δ^3,5^-diene structure. New signal had also appeared in the ^1^H-NMR spectra: the broad triplet at δ 5.47, 5.45, 5.50, 5.42 and 5.39 ppm for H-6 for compounds **2a-e**, respectively. On the other hand, the H-4 signal at δ 6.11, 6.13, 6.14, 6.11 and 6.10 ppm for compounds **2a-e**, respectively, was shifted downfield comparing with that in starting compounds **1a-e** (at δ 5.72, 5.74, 5.84, 5.80¸ 5.70 ppm, respectively). This can be explained by anisotropy effect of the new double bond in the dimers. On the basis of the COSY correlations between the vinylic H-6 and H_2_-7 together with the key HMBC correlations from H-6 to C-7 and C-8 the alternative Δ^2,4^-isomer was ruled out. Furthermore, the elemental analyses for **2a-e** were in agreement with the proposed structures.

**Scheme 2:**
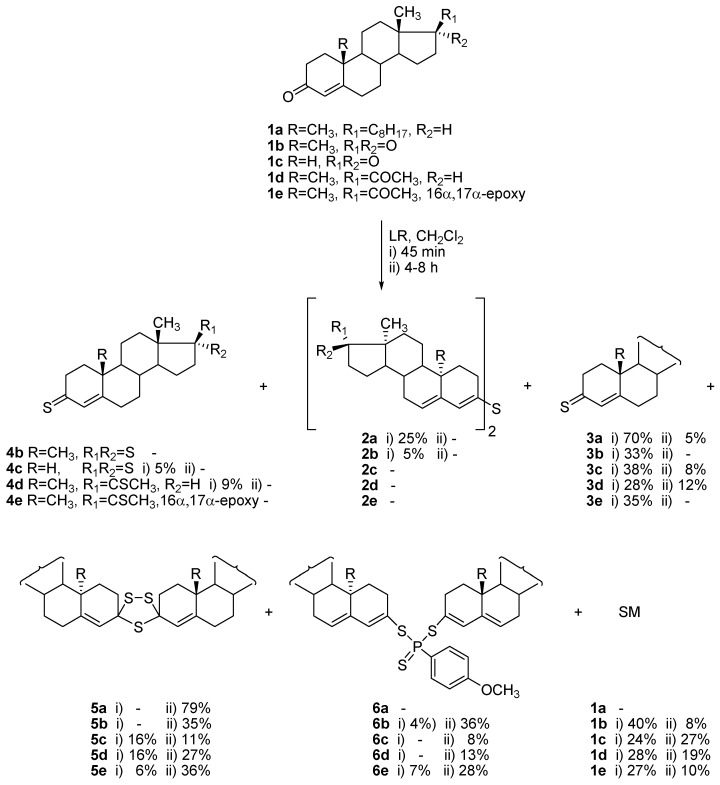
Thionation of **1a**-**e** with LR in CH_2_Cl_2._

In order to increase the yield of thioketones we performed the same reactions under milder conditions, in CH_2_Cl_2_ as a solvent (refluxing for 45 min) and the thioketones **3a**-**e** were obtained in much higher yield, 28–70%. Besides, steroids **1c**-**e** gave corresponding 1,2,4-trithiolanes **5c-e**. The steroids **1b** and **1e** gave also the (4-methoxy-phenyl)phosphonotrithioates **6b** and **6e**. In this reaction the formation of dithioketones **4c** and **4d** was observed as well and these products were also isolated but due to their instability we were able to record only IR spectra in which the absorption for the original unsaturated 3-oxo group was missing as well as the absorptions for carbonyls at C-17 for **4c** and C-20 for **4d** (Scheme 2).

With a prolonged time of reaction in CH_2_Cl_2_ (reflux 4–8 h, depending on substrate, see Experimental) all unsaturated ketones **1a-e** gave as a main product the corresponding 1,2,4-trithiolanes **5a-e** (11–79%). Besides, steroids **1b-e** gave also the (4-methoxyphenyl)phosphonotrithioates **6b-e** (8–36%) as a result of further reaction of firstly formed thioketones with LR. In some cases (**1a**, **1c** and **1d**) the thioketones, **3a**, **3c** and **3d** were still present in the reaction mixture and isolated in very poor yield (5–12%) (Scheme 2).

Support for the structures **5a-e** was found from the ^13^C- and ^1^H-NMR spectral data as well as from the elemental analysis which showed that they contain three additional sulfur atoms (at two steroid molecules). NMR spectral data showed the absence of α,β-unsaturated 3-oxo group. In the ^1^H-NMR spectra signal for H-4 at δ 5.45, 5.44, 5.60, 5.47 and 5.46 ppm for **5a-e**, respectively, was situated upfield comparing with that in the starting compounds **1a-e** (at δ 5.72, 5.74, 5.84, 5.80¸ 5.70 ppm, respectively), demonstrating the effect of an absence of the deshielding influence of 3-oxo group. In the ^13^C-NMR spectra the singlet for C-5 at δ 152.5, 149.8 147.0, 151.9 and 151.8 ppm for **5a-e**, respectively, was also situated upfield when compared to the corresponding resonance in **1a-e**, indicated the absence of α,β-unsaturated carbonyl group. In addition, the singlet for C-3 at about 199 ppm for starting compounds was missing and instead the new singlet at δ 81.3, 80.2, 80.9, 81.1 and 81.1 ppm, respectively for **5a-e**, appeared indicating the formation of 1,2,4-trithiolane ring.

In the IR spectra of phosphonotrithioates **6b-e** the original C(3)=O absorption band disappeared, while in the ^13^C-NMR spectra the singlet for C-3 at about δ 199 ppm was also absent. The ^1^H-NMR spectra showed the new olefinic H-6 proton signal, a broad triplet at δ 5.52, 5.61, 5.49 and 5.47 ppm for **6b-e,** respectively. On the other hand, the resonance for the olefinic H-4 proton appeared like two sets of signals, two doublets at δ 6.30 and 6.34 ppm, 6.38 and 6.40 ppm, 6.28 and 6.32 ppm, 6.28 and 6.31 ppm for compounds **6b-e**, respectively, with *J*_PH_ about 4 Hz indicated two different protons, both with the long range coupling with phosphorus. The signal for 19-Me group also appeared in pairs, two singlets at δ 0.92 and 0.96 ppm for **6b**, 0.89 and 0.92 ppm for **6d** and 0.89 and 0.94 ppm for **6e**. Besides, the new singlet at δ 3.87, 3.86, 3.86 and 3.87 ppm (OCH_3_) appeared as well as two doublets of doublets at δ 6.96 and 8.02, 7.00 and 8.01, 6.96 and 8.02 and 6.95 and 8.01 ppm for aromatic protons for **6b-e,** respectively, and these signals had corresponding *J*_HH_ and *J*_PH_ coupling constants. On basis of the peak areas ratio of the olefinic, methoxy and aromatic protons (H-4/H-6/OCH_3_/ArH(3,5)/ArH(2,6)=2:2:3:2:2) it was deduced that two steroid molecules are attached with one monomeric species of LR to gave (4-methoxyphenyl)phosphonotrithioates **6b**-**e**. The difference between H-4 protons and 19-methyl groups is attributed to the molecular asymmetry. This was confirmed by the ^13^C-NMR spectra in which the C-3 carbon atoms appeared like two doublets at δ 124.5 and 124.8 ppm, 124.7 and 125.2 ppm, 124.4 and 124.5 ppm, 122.4 and 124.7 ppm for **6b-e,** respectively, with ^2^*J*_PC_ about 10 Hz. Furthermore, the signals for C-1, C-2, C-4, C-5, C-6, C-10 and 19-Me appeared like two sets of signals confirming proposed structures. Also, there were the signals for aromatic carbon atoms with corresponding ^1-4^*J*_PC_ coupling constants at δ 162.9, 133.7, 129.6 and 113.9 ppm for compound **6b,** at δ 162.9, 133.4, 129.6, 113.6 ppm for **6c,** at δ 162.6, 133.4, 128.1 and 113.6 ppm for **6d**, and at δ 162.6, 133.4, 127.7 and 113.6 ppm for **6e**.

**Scheme 3:**
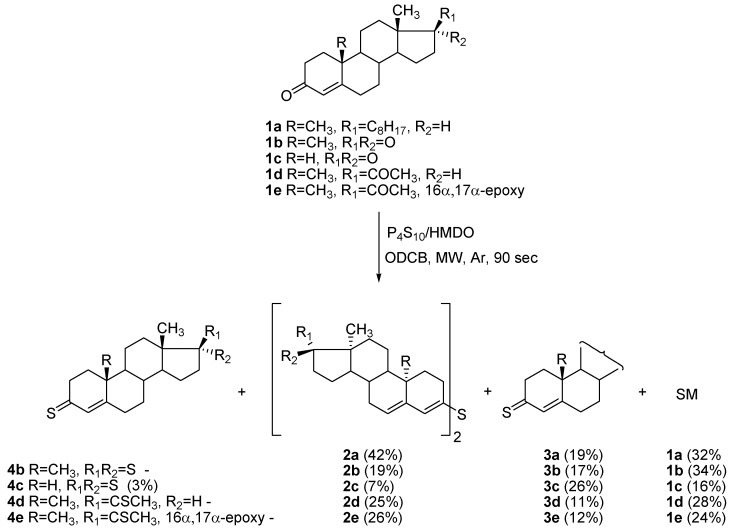
Thionation of **1a**-**e** with P_4_S_10_/HMDO under the microwave irradiation.

Thionation of steroidal ketones **1a-e** with a combination of P_4_S_10_/HMDO under microwave irradiation in all cases gave two products: 3-thioketones **3a-e** (11–26%) and dimer-sulfides **2a-e** (7–42%). The yield of thioketones obtained was lower than in the reaction with LR in CH_2_Cl_2_. In one case, in the reaction of 19-norandrost-4-ene-3,17-dione (**1c**) the 3,17-dithioketone **4c** was isolated as well (3%) (Scheme 3). 

## 3. Experimental Section 

### 3.1. General

Removal of solvents was carried out under reduced pressure. Melting points were determined on an electrothermal capillary melting point apparatus and are uncorrected. IR spectra were recorded on a Perkin-Elmer spectrophotometer FT-IR 1725X: ν in cm^-1^. ^1^H- and ^13^C-NMR spectra were recorded on a Varian Gemini-200 spectrometer (at 200 and 50 MHz, respectively) and/or a Bruker Avance 500 MHz spectrometer (^1^H at 500 MHz; ^13^C at 125 MHz) in CDCl_3_ and/or C_6_D_6_ at room temperature using TMS as internal standard. Chemical shifts are expressed in ppm (δ) values and coupling constants (*J*) in Hz. Mass spectra were taken on Finnigan-MAT 8230. Thin-layer chromatography was performed on precoated Merck silica gel 60 F_254_ plates in toluene/EtOAc (9:1, 8:2) and in *n*-hexane/EtOAc (8:2, 6:4), detection with 50% aq. H_2_SO_4_ and/or with CAM solution. Flash column chromatography (FCC) was performed on silica gel Merck 0.040–0.063 mm under the Ar atmosphere. Elemental analyses were determined on Vario EL *III*. All starting steroid compounds were commercially available products.

### 3.2. General procedure for thionation with LR

LR (1 mmol) was added to a solution of steroidal ketone (1 mmol) in dichloromethane or toluene (15 mL). The reaction mixture was refluxed (duration mentioned in each experiment) with stirring and monitored by TLC. After the completion of reaction the rest of reagent was removed by filtration and evaporated. The residue was chromatographed by FCC using toluene/EtOAc or *n*-hexane/EtOAc (ratio mentioned in each experiment) as eluent.

### 3.3. General procedure for thionation with combination of P_4_S_10_/HMDO under the microwave irradiation

All microwave assisted reactions were carried out using a modified Panasonic NN-S255W domestic microwave oven (2450 MHz, 800 W) which was adapted for laboratory applications with an external reflux condenser. The oven was perforated at the top for a condenser tube and external steel tube of the same diameter (~30 mm) was welded in order to eliminate possible microwave leakage.

P_4_S_10_ (0.5 mmol) was added to a solution of steroidal ketone (2 mmol) in ODCB (5 mL) placed in a 20 mL *quartz vessel*. The reaction mixture was stirred under the argon (Ar) for 10 minutes at room temperature and then HMDO (3.5 mmol) was added. The content was irradiated at 600 W for 90 seconds under Ar atmosphere with a pause of 2 min after every 20 seconds, monitored by TLC. After the completion of reaction the mixture was chromatographed by FCC using toluene/EtOAc or *n*-hexane/EtOAc (ratio mentioned in each experiment) as eluent.

#### 3.3.1. Thionation of cholest-4-en-3-one (**1a**)

*In toluene 45 min*: Starting with 384 mg of **1a,** elution with *n*-hexane gave dimer-sulfide **2a** (216 mg, 56%). Oil. IR (CHCl_3_): 2935, 1465, 732. ^1^H-NMR (500 MHz, CDCl_3_): 0.70 (s, 6H, 2×H_3_C(18)), 0.86 and 0.87 (2d, 12H, 2×H_3_C(26), 2×H_3_C(27)), 0.92 (d, 6H, 2×H_3_C(21)), 0.94 (s, 6H, 2×H_3_C(19)), 5.41 (br.t, *J* = 4.5 Hz, 2H, 2×H-C(6)), 6.11 (2H, 2×H-C(4)). ^13^C-NMR (125 MHz): 141.6 (s, C(5)), 131.7 (d, C(4)), 129.4 (s, C(3)) 124.3 (d, C(6)), 56.9 (d, C(14)), 56.2 (d, C(17)), 48.1 (d, C(9)), 42.5 (s, C(13)), 39.8 (t, C(12)), 39.5 (t, C(24)), 36.2 (t, C(22)), 35.8 (d, C(20), 34.7 (s, C(10)), 34.7 (t, C(1)), 31.9 (t, C(7)), 31.8 (d, C(8)), 28.2 (t, C(16)), 28.0 (d, C(25)), 27.8 (t, C(2)), 24.2 (t, C(15)), 23.8 (t, C(23)), 22.8 (q, C(27)), 22.6 (q, C(26)), 21.1 (t, C(11)), 19.0 (q, C(19)), 18.7 (q, C(21)), 12.0 (q, C(18)). Anal. calcd for C_54_H_86_S: C 84.52; H 11.30; S 4.18. Found: C 84.07; H 11.56; S 4.28. Further elution with *n*-hexane/toluene (70:30) gave 3-thioxocholest-4-ene (**3a**) (45 mg, 11%). Pink oil. IR (CHCl_3_): 2931, 1582, 1024. ^1^H-NMR (200 MHz, C_6_D_6_): 0.61 (s, 3H, H_3_C(18)), 080 (s, 3H, H_3_C(19)), 0.92 (d, 6H, H_3_C(26), H_3_C(27)), 0.97 (d, 3H, H_3_C(21)), 2.50, (dtd, *J*=18.6, 15.3, 4.6, 1H, Hβ-C(2)), 3.11 (dt, *J*=18.0, 3.4, 1H, Hα-C(2)), 6.63 (br.s, 1H, H-C(4)). ^13^C NMR (50 MHz, C_6_D_6_): 236.7 (s, C(3)), 161.8 (s, C(5)), 135.4 (d, C(4)), 56.4 (d, C(14)), 56.0 (d, C(17)) 53.9 (d, C(9)), 43.6 (t, C(2)), 42.6 (s, C(13)), 39.9 (2t, C(12) and C(24)), 38.5 (s, C(10)), 36.7 (t, C(22)), 36.6 (t, C(6)), 36.1 (d, C(20)), 35.7 (d, C(8)), 32.7 (t, C(7)), 32.2 (t, C(1)), 28.5 (t, C(16)), 28.4 (d, C(25)), 24.3 (2t, C(15) and C(23)), 23.0 (q, C(27)), 22.8 (q, C(26)), 21.2 (t, C(11)), 18.9 (q, C(21)), 16.7 (q, C(19)), 12.1 (q, C(18)). Anal. calcd for C_27_H_44_S: C 80.93; H 11.07; S 8.00. Found: C 8.67; H 11.22; S 8.18. Additionally starting material (70 mg, 18%) was recovered.

*In toluene 8 h*: Starting with 384 mg of **1a,** elution with *n*-hexane gave dimer-sulfide **2a** (112 mg, 30%) and starting material (200 mg, 52%).

*In CH_2_Cl_2_ 45 min*: Starting with 380 mg of **1a,** elution with *n*-hexane gave dimer-sulfide **2a** (95 mg, 25%). Further elution with *n*-hexane/toluene (70:30) gave 3-thioxocholest-4-ene (**3a**) (279 mg, 70%). 

*In CH_2_Cl_2_ 4 h*: Starting with 380 mg of **1a**, elution with *n*-hexane and crystallization from MeOH gave 1,2,4-trithiolane **5a** (330 mg, 79% ). m.p. 134–136 ºC. IR (CHCl_3_): 2929, 1464, 733. ^1^H-NMR (200 MHz, CDCl_3_): 0.67 (s, 6H, 2×H_3_C(18)), 086 (d, 12H, 2×H_3_C(26), 2×H_3_C(27)), 0.93 (d, 6H, 2×H_3_C(21)), 1.03 (s, 6H, 2×H_3_C(19)), 5.45 (s, 2H, 2×H-C(4)). ^13^C-NMR: 152.5 (s, C(5)), 119.1 (d, C(4)), 81.3 (s, C(3)), 56.1 (d, C(17)), 55.9 (d, C(14)), 54.4 (d, C(9)), 42.4 (s, C(13)), 39.7 (t, C(12)), 39.5 (t, C(24)), 37.6 (s, C(10)), 37.3 (t, C(2)), 36.1 (t, C(22)), 35.8 (d, C(20)), 35.7 (d, C(8)), 35.0 (t, C(1)), 32.7 (t, C(7)), 32.4 (t, C(6)), 28.2 (t, C(16)), 28.0 (d, C(25)), 24.2 (t, C(15)), 23.8 (t, C(23)), 22.8 (q, C(27)), 22.5 (q, C(26)), 21.2 (t, C(11)), 18.6 (q, C(21)), 18.5 (q, C(19)), 11.9 (q, C(18)). Anal. calcd for C_54_H_88_S_3_: C 77.82; H 10.61; S 11.54. Found: C 77.61; H 10.78; S 11.39. Further elution with *n*-hexane/toluene (70:30) gave 3-thioxocholest-4-ene (**3a**) (20 mg, 5%).

*Under microwave irradiation*: Starting with 762 mg of **1a,** elution with *n*-hexane gave dimer-sulfide **2a** (323 mg, 42%). Further elution with *n*-hexane/toluene (70:30) gave 3-thioxocholest-4-ene (**3a**) (153 mg, 19%). Starting material (244 mg, 32%) was also obtained.

#### 3.3.2. Thionation of androst-4-ene-3,17-dione (**1b**)

*In toluene 45 min*: Starting with 860 mg of **1b**, elution with toluene/EtOAc (97:3) and crystallization from MeOH gave dimer-sulfide **2b** (182 mg, 21%). m.p. > 126 ºC (decomp.). IR (CHCl_3_): 2940, 1737, 732. ^1^H-NMR (200 MHz, CDCl_3_): 0.91 (s, 6H, 2×H_3_C(18)), 0.97 (s, 6H, 2×H_3_C(19)), 5.45 (br. s, 2H, 2×H-C(6)), 6.13 (s, 2H, 2×H-C(4)). ^13^C-NMR: 221.1 (s, C(17)), 141.6 (s, C(5)), 131.4 (d, C(4)), 129.5 (s, C(3)), 123.3 (d, C(6)), 51.7 (d, C(14)), 48.0 (d, C(9)), 47.6 (s, C(13)), 35.7 (t, C(12)), 34.7 (s, C(10)), 34.5 (t, C(1)), 31.3 (t, C(7)), 31.3 (d, C(8)), 30.6 (t, C(16)), 27.6 (t, C(2)), 21.7 (t, C(15)), 20.3 (t, C(11)), 18.9 (q, C(19)), 13.6 (q, C(18)). Anal. calcd for C_38_H_50_O_2_S: C 79.95; H 8.83; S 5.62. Found: C 79.80; H 9.06; S 5.70. Further elution with toluene/EtOAc (95:5) afforded 3-thioxoandrost-4-en-17-one (**3b**) (242 mg, 27%). Pink oil. IR (C_6_D_6_): 2936, 1735, 1579, 1010. ^1^H NMR (200 MHz, C_6_D_6_): 0.63 (s, 3H, H_3_C(18)), 0.74 (s, 3H, H_3_C(19)), 2.52 (dtd, *J*=17.6, 15.6, 5.0, 1H, Hβ-C(2)), 3.15 (dt, *J* = 17.4, 3.4 Hz, 1H, Hα-C(2)), 6.69 (s, 1H, H-C(4)). ^13^C NMR (50 MHz, C_6_D_6_): 237.0 (s, C(3)), 217.7 (s, C(17)), 161.3 (s, C(5)), 135.4 (d, C(4)), 53.7 (d, C(9)), 50.6 (d, C(14)), 47.2 (s, C(13)), 43.5 (t, C(2)), 38.4 (s, C(10)), 36.5 (t, C(16)), 35.5 (t, C(6)), 34.9 (d, C(8)), 32.3 (t, C(7)), 31.7 (t, C(12)), 30.6 (t, C(1)), 21.6 (t, C(15)), 20.3 (t, C(11)), 16.7 (q, C(19)), 13.5 (q, C(18)). Starting material (69 mg, 8%) was also recovered.

*In toluene 8h:* Starting with 572 mg of **1b**, elution with toluene/EtOAc (97:3) and crystallization from MeOH gave dimer-sulfide **2b** (290 mg, 50%) and starring material (217 mg, 38%).

*In CH_2_Cl_2_ 45 min:* Starting with 572 mg of **1b**, elution with toluene/EtOAc (97:3) gave dimer-sulfide **2b** (27 mg, 5%). Further elution with toluene/EtOAc (95:5) afforded 3-thioxoandrost-4-en-17-one (**3b**) (197 mg, 33%). Elution with toluene/EtOAc (93:7) gave phosphonotrithioate **6b** (27 mg, 4%). Starting material (228 mg, 40%) was also obtained.

*In CH_2_Cl_2_ 8 h:* Starting with 572 mg of **1b**, elution with toluene/EtOAc (95:5) and crystallization from MeOH gave 1,2,4-trithiolane **5b** (220 mg, 35%). m.p. 148–150 ºC. IR (CHCl_3_): 2929, 1738, 1439, 732. ^1^H-NMR (200 MHz, CDCl_3_): 0.87 (s, 6H, 2×H_3_C(18)), 1.06 (s, 6H, 2×H_3_C(19)), 5.44 (s, 2H, 2×H-C(4)). ^13^C-NMR: 220.9 (s, C(17)), 149.8 (s, C(5)), 119.2 (d, C(4)), 80.2 (s, C(3)), 53.9 (d, C(9)), 50.9 (d, C(14)), 47.6 (s, C(13)), 37.2 (s, C(10)), 39.7 (t, C(16)), 35.7 (t, C(1)), 35.2 (d, C(8)), 34.9 (t, C(2)), 32.2 (t, C(6)), 31.3 (t, C(7)), 29.6 (t, C(12)), 21.7 (t, C(15)), 20.4 (t, C(11)), 18.5 (q, C(19)), 13.69 (q, C(18)). Anal. calcd for C_38_H_52_O_2_S_3_: C 71.65, H 8.23, S15.10. Found: C 71.19, H 8.27, S 14.84. Further elution with toluene/EtOAc (93:7) gave phosphonotrithioate **6b** (280 mg, 36%). m.p. >133 ºC (decomp.). IR (CHCl_3_): 2936, 1733, 1254, 1096, 828. ^1^H-NMR (500 MHz, CDCl_3_): 0.89 and 0.90 (2s, 6H, 2×H_3_C(18)), 0.92 and 0.96 (2s, 6H, 2×H_3_C(19)), 3.87 (s, 3H, OCH_3_), 5.52 (br.s, 2H, 2×H-C(6)), 6.30 and 6.34 (2d, ^4^*J*_PH_ = 3.0 and 4.5 Hz, 2H, 2×H-C(4)), 6.96 (dd, ^4^*J*_PH_ = 3.5 Hz, *J*_HH_ = 9.0 Hz, 2H, 2×Ar-H), 8.02 (dd, ^3^*J*_PH_ = 14.0 Hz, *J*_HH_ = 9.0 Hz, 2H, 2×Ar-H). ^13^C-NMR (125 MHz): 220.2 (s, C(17)), 162.9 (sd, ^4^*J*_PC_ = 2.9 Hz, Ar*C*-OCH_3_), 142.08 and 142.05 (2sd, ^4^*J*_PC_ = 3.6 and 4.6 Hz, 2×C(5)), 141.46 and 141.38 (2dd, both ^3^*J*_PC_ = 5.5 Hz, 2×C(4)), 133.7 (dd, ^3^*J*_PC_ = 12.5 Hz, 2×ArC-H), 129.6 (sd, *J*_PC_ = 91.8 Hz, ArC-P), 126.83 and 126.80 (2d, 2×C(6)), 124.7 and 124.5 (2sd, ^2^*J*_PC_=12.8 and 9.1, 2×C(3)), 113.9 (dd, ^2^*J*_PC_=15.0, 2×ArC-H), 55.7 (q, O*C*H_3_), 52.0 (d, C(14)), 48.2 (d, C(9)), 47.9 (s, C(13)), 36.0 (t, C(16)), 35.0 and 34.9 (2t, 2×C(1)), 34.73 and 34.70 (2s, 2×C(10)), 31.6 (d, C(7)), 31.5 (t, C(8)), 31.4 and 31.2, 2t, 2×C(2)), 31.06 (t, C(12)), 22.0 (t, C(15)), 20.6 (t, C(11)), 19.16 and 19.22 (2q, 2×C(19)), 13.9 (q, C(18)). ESI-TOF-MS: *m/z* calcd for C_45_H_57_O_3_PS_3_: 773.3286 [M+H]^+^, found 773.2404. Starting material (46 mg, 8%) was recovered too.

*Under microwave irradiation:* Starting with 572 mg of **1b**, elution with toluene/EtOAc (97:3) gave dimer-sulfide **2b** (105 mg, 19%). Further elution with toluene/EtOAc (95:5) afforded 3-thioxoandrost-4-en-17-one (**3b**) (100 mg, 17%). Starting material (195 mg, 34%) was isolated.

#### 3.3.3. Thionation of 19-norandrost-4-ene-3,17-dione (**1c**)

*In toluene 25 min:* Starting with 540 mg of **1c**, elution with toluene/EtOAc (98:2) afforded dimer-sulfide **2c** (37 mg, 7%). IR (CHCl_3_): 2928, 1737. ^1^H-NMR (200 MHz, CDCl_3_): 0.92 (s, 6H, 2×H_3_C(18)), 5.55 (br. s, 2H, 2×H-C(6)), 6.14 (s, 2H, 2×H-C(4)). ^13^C-NMR: 221.01 (s, C(17)), 141.6 (s, C(5)), 131.4 (d, C(4)), 129.5 (s, C(3)), 124.8 (d, C(6)), 50.1 (d, C(14)), 49.4 (d, C(9)), 47.7 (s, C(13)), 42.4 (s, C(10)), 42.4 (d, C(8)), 36.4 (t, C(16)), 31.3 (t, C(7)), 30.6 (t, C(12)), 29.6 (t, C(2)), 26.5 (t, C(1)), 25.6 (t, C(11)), 21.7 (t, C(15)), 13.6 (q, C(18)). Anal. calcd for C_36_H_36_O_2_S: C 79.66; H 8.54; S 5.91. Found: C 79.80; H 8.72; S 6.00. Further elution with toluene/EtOAc (97:3) afforded 3-thioxo-19-norandrost-4-en-17-one (**3c**) (140 mg, 25%). Pink oil. IR (C_6_D_6_): 2929, 1731, 1560, 1034. ^1^H-NMR (500 MHz, C_6_D_6_): 0.60 (s, 3H, H_3_C(18)), 2.35 (dtd, *J* = 15.8, 14.5, 4.5 Hz, 1H, Hβ-C(2)), 3.15 (dt, *J* = 16.5, 4.0 Hz, 1H, Hα-C(2)), 6.75 (s, 1H, H-C(4)). ^13^C-NMR (125 MHz, C_6_D_6_): 237.8 (s, C(3)), 217.8 (s, C(17)), 156.9 (s, C(5)), 136.6 (d, C(4)), 50.3 (d, C(14)), 49.7 (d, C(9)), 47.8 (s, C(13)), 46.5 (t, C(2)), 42.9 (d, C(10)), 40.1 (d, C(8), 35.9 (t, C(16)), 35.4 (t, C(6)), 32.1(t, C(7)), 30.2 (t, C(12)), 27.9 (t, C(1)), 26.0 (t, C(15)), 21.9 (t, C(11)), 14.0 (q, C(18)). Anal. calcd for C_18_H_24_OS: C 74.95; H 8.39; S 11.12. Found: C 75.10; H 8.68; S 11.27. Starting material (200 mg, 37%) was isolated.

*In toluene 8 h:* Starting with 540 mg of **1c**, elution with toluene/EtOAc (98:2) afforded dimer-sulfide **2c** (230 mg, 43%) and starting material (140 mg, 26%).

*In CH_2_Cl_2_ 45 min:* Starting with 544 mg of **1c**, elution with toluene/EtOAc (99:1) gave 3,17-dithioxo-19-norandrost-4-ene (**4c**) (29 mg, 5%). IR (KBr): 2845, 1582. Further elution with toluene/EtOAc (97:3) afforded 3-thioxo-19-norandrost-4-en-17-one (**3c**) (220 mg, 38%). Further elution with toluene/EtOAc (95:5) gave 1,2,4-trithiolane **5c** (99 mg, 16%). IR (CHCl_3_): 2928, 1737, 1403. ^1^H-NMR (200 MHz, CDCl_3_): 0.90 (s, 6H, 2×H_3_C(18)), 5.60 (s, 2H, 2×H-C(4)). ^13^C-NMR: 221.0 (s, C(17)), 147.0 (s, C(5)), 120.5 (d, C(4)), 80.9 (s, C(3)), 50.1 (d, C(9)), 50.1 (d, C(14)), 47.7 (s, C(13)), 41.3 (d, C(10)), 39.9 (d, C(8)), 37.5 (t, C(2)), 36.6 (t, C(16)), 34.8 (t, C(6)), 31.3 (t, C(7)), 30.1 (t, C(12)), 28.0 (t, C(1)), 25.4 (t, C(11)), 21.5 (t, C(15)), 13.7 (q, C(18)). Anal. calcd for C_36_H_48_O_2_S_3_: C 71.00; H 7.94; S 15.80. Found: C 71.42; H 7.88; S 15.56. Starting material recovery: 130 mg, (24%).

*In CH_2_Cl_2_ 7 h:* Starting with 544 mg of **1c**, elution with toluene/EtOAc (97:3) afforded 3-thioxo-19-norandrost-4-en-17-one (**3c**) (47 mg, 8%). Further elution with toluene/EtOAc (95:5) gave 1,2,4-trithiolane **5c** (65 mg, 11%). Further elution with toluene/EtOAc (94:6) gave phosphonotrithioate **6c** (59 mg, 8%). IR (CHCl_3_):2929, 1730, 1439, 1096, ^1^H-NMR (200 MHz, CDCl_3_): 0.89 (s, 6H, 2×H_3_C(18)), 3.86 (s, 3H, OCH_3_), 5.61 (br.s, 2H, 2×H-C(6)), 6.38 and 6.40 (2d, both ^4^*J*_PH_ = 4.4 Hz, 2H, 2×H-C(4)), 7.00 (dd, ^4^*J*_PH_ = 3.4 Hz, *J*_HH_ = 8.4 Hz, 2H, 2×Ar-H), 8.01 (dd, ^3^*J*_PH_ = 14.0 Hz, *J*_HH_ = 9.0 Hz, 2H, 2×Ar-H). ^13^C-NMR(50 MHz): 220.9 (s, C(17)), 162.9 (s, Ar*C*-OCH_3_) 142.1 (s, C(5)), 141.6 and 141.7 (2d, 2×C(4)), 133.4 (dd, ^3^*J*_PC_ = 12.8 Hz, 2×ArC-H), 129.6 (sd, *J*_PC_ = 91.8 Hz, ArC-P), 126.9 (d, C(6)), 125.2 and 124.7 (2sd, both ^2^*J*_PC_ = 12.0 Hz, 2×C(3)), 113.6 (dd, ^2^*J*_PC_ = 14.6 Hz, 2×ArC-H), 55.4 (q, O*C*H_3_), 50.8 (d, C(14)), 49.9 (d, C(9)), 47.7 (s, C(13)), 43.5 (d, C(10)), 39.9 (d, C(8)), 35.7 (t, C(16)), 31.2 (2t, C(2) and C(7)), 30.3 (t, C(12)), 28.0 (t, C(1)), 25.4 (t, C(11)), 21.4 (t, C(15)), 13.7 (q, C(18)). Starting material (147 mg, 27%) was also obtained.

*Under the microwave* *irradiation:* Starting with 540 mg of **1c**, elution with toluene/EtOAc (99:1) gave 3,17-dithioxo-19-norandrost-4-ene (**4c**) (18 mg, 3%). IR: 2845, 1582, 1035. Further elution with toluene/EtOAc (98:2) afforded dimer-sulfide **2c** (38 mg, 7%). Further elution with toluene/EtOAc (97:3) afforded 3-thioxo-19-norandrost-4-en-17-one (**3c**) (149 mg, 26%). Starting material (86 mg, 16%) was isolated.

#### 3.3.4. Thionation of progesterone (**1d**)

*In toluene 45 min:* Starting with 314 mg of **1d**, elution with toluene/EtOAc (99:1) gave 3-thioxoprogesterone (**3d**) (73 mg, 22%). Pink oil. IR (C_6_D_6_): 2936, 1702, 1579, 1010. ^1^H-NMR (200 MHz, C_6_D_6_): 0.54 (s, 3H, H_3_C(18)), 076 (s, 3H, H_3_C(19)), 1.86 (s, 3H, H_3_C(21)), 2.52 (dtd, *J* = 16.5, 15.5, 5.0 Hz, 1H, Hβ-C(2)), 3.14 (dt, *J* = 17.8, 3.6 Hz, 1H, Hα-C(2), 6.66 (s, 1H, H-C(4)). ^13^C-NMR (50 MHz, C_6_D_6_): 236.9 (s, C(3)), 206.9 (s, C(20)), 161.9 (s, C(5)), 135.4 (d, C(4)), 63.3 (d, C(17)), 55.9 (d, C(14)), 53.7 (d, C(9)), 43.7 (s, C(13)), 43.6 (t, C(2)), 38.6 (t, C(12)), 38.4 (s, C(10)), 36.6 (t, C(6)), 35.4 (d, C(8)), 32.6 (t, C(7)), 31.9 (t, C(1)), 31.1 (q, C(21)), 24.4 (t, C(15)), 23.1 (t, C(16)), 21.1 (t, C(11)), 16.8 **(**q, C(19)), 13.4 (q, C(18)). Further elution with toluene/EtOAc (98:2) afforded dimer-sulfide **2d** (50 mg, 16%). IR (CHCl_3_): 2939, 1703, 1257. ^1^H-NMR (200 MHz, CDCl_3_): 0.65 (s, 6H, 2×H_3_C(18)), 0.94 (s, 6H, 2×H_3_C(19)), 2.13 (s, 6H, 2×H_3_C(21)), 5.42 (br. s, 2H, 2×H-C(6)), 6.11 (s, 2H, 2×H-C(4)). ^13^C-NMR: 209.4 (s, C(20)), 141.4 (s, C(5)), 131.6 (d, C(4)), 129.3 (s, C(3)), 123.8 (d, C(6)), 63.5 (d, C(17), 56.9 (d, C(14)), 47.8 (d, C(9)), 44.0 (s, C(13)), 38.7 (t, C(12)), 34.6 (s, C(10)), 34.6 (t, C(1)), 31.6 (d, C(8)), 31.6 (q, C(21)), 31.4 (t, C(7)), 27.6 (t, C(2)), 24.3 (t, C(15)), 22.7 (t, C(16)), 21.0 (t, C(11)), 18.9 (q, C(19)), 13.2 (q, C(18)). Anal. calcd for C_42_H_58_O_2_S: C 80.46; H 9.32; S 5.11. Found: C 80.68; H 9.41; S 13.69. Starting material recovered: 119 mg (38%).

*In toluene 8 h:* Starting with 314 mg of **1d**, elution with toluene/EtOAc (98:2) gave dimer-sulfide **2d** (160 mg, 51%) and starting material (106 mg, 34%).

*In CH_2_Cl_2_ 45 min:*Starting with 630 mg of **1d**, elution with toluene/EtOAc (99:1) gave 3,20-dithioxoprogesterone (**4d**) (66 mg, 9%). IR (KBr): 2939, 1576, 1010. Further elution with toluene/EtOAc (98:2) afforded 3-thioxoprogesterone (**3d**) (185 mg, 28%). Further elution with toluene/EtOAc (97:3) gave 1,2,4-trithiolane **5d** (114 mg, 16%). IR (CHCl_3_): 2934, 1702, 1492. ^1^H- NMR (200 MHz, CDCl_3_): 0.63 (s, 6H, 2×H_3_C(18)), 1.03 (s, 6H, 2×H_3_C(19)), 2.13 (s, 6H, 2×H_3_C(21)), 5.47 (s, 2H, 2×H-C(4)). ^13^C-NMR: 209.5 (s, C(20)), 151.9 (s, C(5)), 119.3 (d, C(4)), 81.1 (s, C(3)), 63.5 (d, C(17), 56.0 (d, C(14)), 54.2 (d, C(9)), 44.0 (s, C(13)), 38.7 (t, C(12)), 37.2 (s, C(10)), 37.2 (t, C(2)), 35.6 (d, C(8)), 34.9 (t, C(1)), 32.5 (t, C(6)), 32.1 (t, C(7)), 31.4 (q, C(21)), 24.3 (t, C(15)), 22.7 (t, C(16)), 21.1 (t, C(11)), 16.5 **(**q, C(19)), 13.2 (q, C(18)). Anal. calcd for C_42_H_60_O_2_S_3_: C 72.78; H 8.73; S 13.88. Found: C 72.91; H 8.67; S 13.80. Recovered starting material: 176 mg (28%).

*In CH_2_Cl_2_ 7 h:* Starting with 630 mg of **1d**, elution with toluene/EtOAc (99:1) gave 3-thioxoprogesterone (**3d**) (78 mg, 12%). Further elution with toluene/EtOAc (97:3) gave 1,2,4-trithiolane **5d** (185 mg, 27%). Further elution with toluene/EtOAc (96:4) gave phosphonotrithioate **6d** (110 mg, 13%). IR (CHCl_3_): 2936, 1702, 1257, 1098, 830. ^1^H-NMR (500 MHz, CDCl_3_): 0.637 and 0.640 (2s, 6H, 2×H_3_C(18)), 0.89 and 0.92 (2s, 6H, 2×H_3_C(19)), 2.117 and 2.120 (2s, 6H, 2×H_3_C(21)), 3.86 (s, 3H, OCH_3_), 5.49 (br.s, 2H, 2×H-C(6)), 6.28 and 6.32 (2d, ^4^*J*_PH_ = 4.5 and 6.0 Hz, 2H, 2×H-C(4)), 6.96 (dd, ^4^*J*_PH_ = 3.2 Hz, *J*_HH_ = 9.0 Hz, 2H, 2×Ar-H), 8.02 (dd, ^3^*J*_PH_ = 14.0 Hz, *J*_HH_ = 9.0 Hz, 2H, 2×Ar-H). ^13^C NMR (125 MHz): 209.4 (s, C(20)), 162.6 (sd, ^4^*J*_PC_ = 2.9 Hz, Ar*C*-OCH_3_), 141.6 and 141.7 (2sd, both ^4^*J*_PC_ = 3.7 Hz, 2×C(5)), 141.27 and 141.35 (2dd, ^3^*J*_PC_ = 5.5 Hz, 2×C(4)), 133.4 (dd, ^3^*J*_PC_ = 12.8 Hz, 2×ArC-H), 128.1 (sd, *J*_PC_ = 84.6 Hz, ArC-P), 127.23 and 127.20 (2dd, ^5^*J*_PC_ = 3.7 and 5.0 Hz, 2×C(6)), 124.5 and 124.4 (2sd, ^2^*J*_PC_ = 8.7 and 10.0 Hz, 2×C(3), 113.6 (d, ^2^*J*_PC_ = 15.0 Hz, 2×ArC-H)), 63.5 (d, C(17), 56.9 (d, C(9)), 55.5 (q, OCH_3_), 47.7 (d, C(14)), 44.1 (s, C(13)), 38.7 (t, C(12)), 34.80 and 34.78 (2t, 2×(C(1)), 34.34 and 34.31 (2s, 2×C(10)), 31.8 (t, C(7)), 31.6 (d, C(8)), 31.5 (q, C(21)), 31.3 and 31.1 (2t, 2×C(2)), 24.2 (t, C(15)), 22.6 (t, C(16)), 20.9 (t, C(11)), 18.93 and 18.88 (2q, 2×C(19)), 13.3 (q, C(18)). ESI-TOF-MS: *m/z* calcd for C_49_H_65_O_3_PS_3_: 829.39062 [M+H]^+^, found 829.38920. In addition starting material (120 mg, 19%) was isolated.

*Under microwave irradiation:* Starting with 630 mg of **1d**, elution with toluene/EtOAc (99:1) gave 3-thioxoprogesterone (**3d**) (72 mg, 11%). Further elution with toluene/EtOAc (98:2) afforded gave dimer-sulfide **2d** (152 mg, 25%). Starting material (158 mg, 28%) was also recovered.

#### 3.3.5. Thionation of 16α,17α-epoxyprogesterone (**1e**)

*In toluene 25 min:* Starting with 500 mg of **1e**, elution with toluene/EtOAc (99:1) gave 3-thioxo-16α,17α-epoxyprogesterone (**3e**) (105 mg, 20%). Pink oil. IR (C_6_D_6_): 2942, 1701, 1574, 1075. ^1^H- NMR (500 MHz, C_6_D_6_): 0.67 (s, 3H, H_3_C(18)), 098 (s, 3H, H_3_C(19)), 1.77 (s, 3H, H_3_C(21)), 2.44 (dtd, *J* = 16.2, 15.8, 5.0 Hz, 1H, Hβ-C(2)), 3.04 (s, H-C(16)), 3.09 (dt, *J* = 17.5, 3.5 Hz, 1H, Hα-C(2)), 6.63 (s, 1H, H-C(4)). ^13^C-NMR (125 MHz, C_6_D_6_): 237.4 (s, C(3)), 204.0 (s, C(20)), 161.3 (s, C(5)), 135.8 (d, C(4)), 71.1 (s, C(17)), 60.4 (d, C(16)), 54.1 (d, C(9)), 45.4 (d, C(14)), 43.9 (t, C(2)), 42.1 (s, C(13)), 38.8 (t, C(12)), 38.8 (s, C(10)), 33.6 (d, C(8)), 32.8 (t, C(6)), 31.9 (t, C(7)), 31.8 (t, C(1)), 27.7 (t, C(15)), 25.9 (q, C(21)), 21.8 (t, C(11)), 17.0 **(**q, C(19)), 15.7 (q, C(18)). Further elution with toluene/EtOAc (98:2) afforded gave dimer-sulfide **2e** (122 mg, 25%). IR (CHCl_3_): 2939, 1703, 1376. ^1^H-NMR (200 MHz, CDCl_3_): 0.95 (s, 6H, 2×H_3_C(19)), 1.06 (s, 6H, 2×H_3_C(18)), 2.02 (s, 6H, 2×H_3_C(21)), 3.68 (s, 2H, 2×H-C(16)), 5.39 (br. s, 2H, 2×H-C(6)), 6.10 (s, 2H, 2×H-C(4)). ^13^C-NMR (50 MHz, CDCl_3_): 204.8 (s, C(20)), 141.6 (s, C(5)), 131.4 (d, C(4)), 129.4 (s, C(3)), 123.3 (d, C(6)), 70.8 (s, C(17)), 60.3 (d, C(16)), 48.1 (d, C(14)), 45.4 (d, C(9)), 41.5 (s, C(13)), 34.7 (s, C(10)), 34.4 (t, C(1)), 31.2 (t, C(12)), 31.1 (t, C(2)), 31.1 (t, C(7)), 29.4 (d, C(8)), 27.2 (t, C(15)), 25.8 (q, C(21)), 21.3 (t, C(11)), 18.8 (q, C(19)), 15.1 (q, C(18)). Anal. calcd for C_42_H_54_O_4_S: C 77.02; H 8.31; S 4.90. Found: C 77.38; H 8.53; S 5.10. Starting material recovered: 180 mg (36%).

*In toluene 8 h:* Starting with 500 mg of **1e,** elution with toluene/EtOAc (98:2) afforded gave dimer-sulfide **2e** (202 mg, 41%) and starting material (155 mg, 31%).

*In CH_2_Cl_2_ 45 min:* Starting with 500 mg of **1e**, elution with toluene/EtOAc (99:1) gave 3-thioxo-16α,17α-epoxyprogesterone (**3e**) (185 mg, 35%). Further elution with toluene/EtOAc (98:2) and crystallization from CH_3_OH afforded 1,2,4-trithiolane **5e** (62 mg, 6%). m.p. > 147 ºC (decomp.). IR (CHCl_3_): 2936, 1705, 1377, 733. ^1^H-NMR (200 MHz, CDCl_3_): 1.03 (s, 6H, 2×H_3_C(19)), 1.04 (s, 6H, 2×H_3_C(18)), 2.02 (s, 6H, 2×H_3_C(21)), 3.66 (s, 2H, 2×H-C(16)), 5.46 (s, 2H, 2×H-C(4)). ^13^C-NMR (50 MHz, CDCl_3_): 204.9 (s, C(20)), 151.8 (s, C(5)), 119.5 (d, C(4)), 81.1 (s, C(3)), 70.8 (s, C(17)), 60.4 (d, C(16)), 54.5 (d, C(9)), 44.8 (d, C(14)), 41.6 (s, C(13)), 37.3 (t, C(2)), 37.3 (s, C(10)), 34.9 (t, C(1)), 33.3 (d, C(8)), 32.2 (t, C(6)), 32.2 (t, C(7)), 31.2 (t, C(12)), 27.3 (t, C(15)), 25.9 (q, C(21)), 20.5 (t, C(11)), 18.3 (q, C(19)), 15.2 (q, C(18)). Anal. calcd for C_42_H_56_O_4_S_3_: C 69.96; H 7.83; S 13.34. Found: C 69.68; H 8.11; S 13.47. Further elution with same eluent gave phosphonotrithioate **6e** (42 mg, 7%). Oil. IR (CHCl_3_): 2941, 1701, 1135, 984, 723. ^1^H-NMR (200 MHz, CDCl_3_): 0.89 and 0.94 (2s, 6H, 2×H_3_C(19)), 1.05 (s, 6H, 2×H_3_C(18)), 2.02 (s, 6H, 2×H_3_C(21)), 3.69 (s, 2H, 2×H-C(16)), 3.87 (s, 3H, OCH_3_), 5.47 (br.s, 2H, 2×H-C(6)), 6.28 and 6.31 (2sd, ^4^*J*_PH_ = 5.8 and 6.0 Hz, 2H, 2×H-C(4)), 6.95 (dd, ^4^*J*_PH_ = 3.0 Hz, *J*_HH_ = 8.8 Hz, 2H, 2×Ar-H), 8.01 (dd, ^3^*J*_PH_ = 14.0 Hz, *J*_HH_ = 8.8 Hz, 2H, 2×Ar-H). ^13^C- NMR (50MHz, CDCl_3_): 204.8 (s, C(20)), 162.6 (s, Ar*C*-OCH_3_), 141.85 and 141.78 (2s, C(5)), 141.3 and 141.1 (2d, 2×C(4)), 133.4 (dd, ^3^*J*_PC_ = 12.8 Hz, 2×ArC-H), 127.7 (sd, *J*_PC_ = 84.7 Hz, ArC-P), 126.7 (d, C(6)), 124.7 and 124.4 (2sd, both ^2^*J*_PC_ = 9.1 Hz, 2×C(3)), 113.6 (dd, ^2^*J*_PC_=14.6, 2×ArC-H), 70.8 (s, C(17)), 60.3 (d, C(16)), 55.4 (q, O*C*H_3_), 47.9 (d, C(9)), 45.4 (d, C(14)), 41.5 (s, C(13)), 34.6 (t, C(1)), 34.4 (s, C(10)), 31.4 (t, C(12)), 31.2 (t, C(7)), 31.0 (t, C(2)), 29.3 (d, C(8)), 27.3 (t, C(15)), 25.8 (q, C(21)), 20.3 (t, C(11)), 18.8 (q, C(19)), 15.1 (q, C(18)). Starting material obtained: 135 mg (27%).

*In CH_2_Cl_2_ 8h:* Starting with 500 mg of **1e**, elution with toluene/EtOAc (98:2) gave 1,2,4-trithiolane **5e** (200 mg, 36%). Further elution with same eluent gave phosphonotrithioate **6e** (180 mg, 28%). Starting material: 50 mg (10%).

*Under microwave irradiation:* Starting with 500 mg of **1e**, elution with toluene/EtOAc (99:1) gave 3-thioxo-16α,17α-epoxyprogesterone (**3e**) (63 mg, 12%). Further elution with toluene/EtOAc (98:2) afforded dimer-sulfide **2e** (127 mg, 26%). Starting material isolated: 120 mg (24%).

## 4. Conclusions 

In this work we showed that Lawesson’s reagent can be suitable for preparation of α,β-unsaturated steroidal thioketones as well as different, depending on the solvent, sulfur steroid derivatives. α,β-Unsaturated ketones **1a-e** in boiling toluene gave, besides dimer-sulfides **2a**-**e,** the thioketones **3a**-**e** in 11–27% yields. Much higher yields of thioketones were obtained when thionation was carried out in CH_2_Cl_2_ (28–70%). In this reaction the dithioketones **4c** and **4d** were isolated as well. With prolonged reaction times in both solvents and due to the enethiones instability and formation of by-products neither thioketone could be isolated. In toluene, the initially formed unstable enethiones dimerize to give only the corresponding dimer-sulfides **2a-e** (30–51%). In CH_2_Cl_2_ all unsaturated ketones **1a-e** gave as a main product the corresponding dimers with 1,2,4-trithiolane ring **5a-e** in 11-79% yields, and (4-methoxyphenyl)phosphonotrithioates **6b-e** (8–36%) as a result of further reaction of the initially formed thioketones with LR.

The combination of P_4_S_10_/HMDO under the microwave irradiation can also be applied for thionation of α,β-unsaturated steroids. This reaction gave two main products, 3-thioketones **3a-e** and dimer-sulfides **2a-e**. However, the yields of synthesized thioketones (11–26%) were lower than in the reaction with LR

Although all obtained products were separated using flash column chromatography partial decomposition to the more stable starting α,β-unsaturated ketones took place in all cases. The synthetic results of this work could be useful for chemists in general, not only those working in the field of steroid chemistry. It could be used for preparation of α,β-unsaturated thioketones as well as new sulfur and/or phosphorus containing compounds.
